# The epidemiology of pediatric oncology and hematopoietic cell transplant admissions to U.S. intensive care units from 2001-2019

**DOI:** 10.3389/fonc.2024.1501977

**Published:** 2024-12-03

**Authors:** Kyle B. Lenz, R. Scott Watson, Jennifer J. Wilkes, Matthew R. Keller, Mary E. Hartman, Elizabeth Y. Killien

**Affiliations:** ^1^ Division of Pediatric Critical Care Medicine, Department of Pediatrics, Seattle Children’s Hospital, University of Washington, Seattle, WA, United States; ^2^ Center for Child Health, Behavior & Development, Seattle Children’s Research Institute, Seattle, WA, United States; ^3^ Division of Hematology/Oncology, Department of Pediatrics, Seattle Children’s Hospital, University of Washington, Seattle, WA, United States; ^4^ Institute for Informatics, Washington University in St Louis, St Louis, MO, United States; ^5^ Division of Pediatric Critical Care Medicine, Department of Pediatrics, Washington University in St Louis, St Louis, MO, United States

**Keywords:** pediatric, critical care, hematopoietic stem cell transplant, oncology, epidemiology

## Abstract

Children with cancer or hematopoietic cell transplant (HCT) frequently require ICU care. We conducted a retrospective cohort study using Healthcare Cost and Utilization Project’s State Inpatient Databases from 21 U.S. states from 2001-2019. We included children <18 years with oncologic or HCT diagnosis and used ICD-9-CM and ICD-10-CM codes to identify diagnoses, comorbidities, and organ failures. We used generalized linear Poisson regression and Cuzick’s test of trend to evaluate changes from 2001-2019. Among 2,157,991 total pediatric inpatient admissions, 3.9% (n=82,988) were among oncology patients and 0.3% (n=7,381) were among HCT patients. ICU admission prevalence rose from 13.6% in 2001 to 14.4% in 2019 for oncology admissions and declined from 23.9% to 19.5%, for HCT admissions. Between 2001-2019, the prevalence of chronic non-oncologic comorbidities among ICU patients rose from 44.3% to 69.1% for oncology patients (RR 1.60 [95% CI 1.46-1.66]) and from 41.4% to 81.5% (RR 1.94 [95% CI 1.61-2.34]) for HCT patients. The risk of Multiple Organ Dysfunction Syndrome more than tripled for oncology (9.5% to 33.3%; RR 3.52 [95% CI 2.97-4.18]) and HCT (12.4% to 39.7%; RR 3.20 [95% CI 2.09-4.89]) patients. Mortality decreased most for ICU patients with acute myeloid leukemia (AML) (14.6% to 8.5%) and oncology-related HCTs (15.5% to 9.2%). Critically ill pediatric oncology and HCT patients are increasingly medically complex with greater prevalence of chronic comorbidities and organ failure, but mortality did not increase. Pediatric ICUs may require increased financial and staffing support to care for these patients in the future.

## Introduction

Children with cancer frequently require pediatric intensive care unit (PICU) admission during their treatment course with PICU admission prevalence as high as 46% for some diagnoses (1–3). In some cohorts, the percentage of oncology patients admitted to a PICU has increased in recent years (4, 5). Children who undergo hemopoietic stem cell transplant (HCT) also have a high PICU admission prevalence, ranging from 15-39% (6, 7). Pediatric oncology patients admitted to the PICU have high rates of organ dysfunction and support needs (8–10), and HCT patients may experience additional complications including sinusoidal obstructive syndrome, graft versus host disease, transplant-associated thrombotic microangiopathy, and transplant-related respiratory failure (11–14). In addition to high morbidity rates, mortality compared to the general PICU population is three-fold higher for oncology patients (1, 9, 15) and eight-fold higher for HCT patients (16, 17).

Little is known about how patient characteristics, organ support, and outcomes have changed over time across the entire spectrum of ICUs that care for children with cancer or HCTs. One study in 36 children’s hospitals demonstrated that pediatric oncology patients admitted to PICUs received increased organ support between 2012-2021 (5), but no studies have included care occurring outside of academic PICUs. It is unknown how commonly children with oncologic conditions or HCTs are receiving care outside of children’s hospitals or academic PICUs. Additionally, no studies have compared ICU admissions, organ support, or outcomes between types of oncologic diagnoses. Better understanding of these facets of critically ill pediatric oncology and HCT populations could inform health care delivery and resource allocation by adjusting surveillance for high-risk groups and directing future research priorities.

We analyzed a nationally-representative dataset including all hospital admissions from 21 U.S. states to assess how ICU admission frequency, patient and hospital characteristics, organ dysfunction and support, and outcomes have changed for pediatric oncology and HCT patients from 2001 to 2019, and compared these factors across oncologic diagnoses. We hypothesized that, consistent with general PICU trends (18), ICU admission frequency, prevalence of chronic comorbid conditions, and organ dysfunction have all increased among pediatric oncology and HCT patients over the past two decades.

## Materials and methods

### Study design

We conducted a retrospective population-based cohort study using the Healthcare Cost and Utilization Project’s (HCUP) State Inpatient Databases (SIDs) from 21 geographically disperse U.S. states in 2001, 2004, 2010, 2016, and 2019 (**Supplementary Table 1**). The SIDs include inpatient records for all discharges from non-federal acute care hospitals within each state. We included states that submitted SIDs to HCUP with revenue codes allowing identification of ICU care, and we followed the Strengthening the Reporting of Observational Studies in Epidemiology (STROBE) reporting guideline (19). The study was determined to be exempt from human subject review by the Seattle Children’s Hospital Institutional Review Board.

### Participants

We included all children aged 0-17 years, excluding those in major diagnostic categories 14 (delivering a baby) and 15 (newborns and other neonates) and those admitted to rehabilitation and psychiatric hospitals. We identified ICU care using revenue codes and included admissions to any non-neonatal ICU in the analyses.

### Exposures and outcomes

To evaluate representation compared to the general ICU population, we obtained patient characteristics including age, sex, race, ethnicity, and insurance status as reported by individual hospitals. We used *International Classification of Diseases, Ninth and Tenth Revision* (ICD-9 and ICD-10) codes to identify diagnoses, comorbid conditions, organ failures, and procedures (**Supplementary Table 2**). We categorized admissions into those involving hematologic malignancies, solid malignancies, and HCTs, as well as specific oncologic diagnoses and oncologic versus non-oncologic indications for HCT. We classified comorbidities using the Pediatric Complex Chronic Conditions classification system (20), excluding codes for hematologic or oncologic comorbidities. Technology dependence per the classification system includes presence of devices such as tracheostomies, gastrostomies, colostomies, ventricular shunts, dialysis access, and pacemakers. Metabolic comorbidities included endocrine, amino acid metabolism, lipid metabolism, and storage disorders. We identified organ failures using ICD-9 and ICD-10 codes per previously published algorithms (21, 22) and considered patients to have multiple organ dysfunction syndrome (MODS) if they had two or more dysfunctional organ systems. We estimated total hospital costs using hospital-specific cost-to-charge ratios adjusted to 2019 dollars using the Consumer Price Index (23).

### Statistical analysis

We summarized categorical variables using percentages and continuous variables using medians and interquartile ranges (IQR). We determined the population-based rate of inpatient and ICU admissions per 1000 children using U.S. census data from each included year and calculated incidence rate ratios (IRR) for each year relative to 2001. We used generalized linear Poisson regression to estimate the relative risk (RR) of each categorical variable in each year relative to 2001 and linear regression to estimate mean change in each year relative to 2001 for continuous variables. We used Cuzick’s test of trend to determine overall trends from 2001-2019. We conducted all analyses using Stata version 17 (StataCorp LLC, College Station, TX).

## Results

### Cohort description

In all study years combined, there were 2,157,991 pediatric hospital admissions, of which 12.8% (n=275,656) included ICU care. There were 82,988 admissions for children with oncologic diagnoses (3.8% of hospital admissions) and 7,381 admissions for children who had received an HCT (0.3% of hospital admissions) (**Table 1**). A total of 13.9% (n=11,517) of oncologic admissions and 23.7% (n=1,749) of HCT admissions included ICU admission.

**Table 1:** Demographic traits of pediatric oncology and hematopoietic cell transplant patients admitted to U.S. intensive care units.

Patient characteristic	2001	2004	2010	2016	2019	p-value
All pediatric hospitalizations, No.	295,857	351,929	602,867	536,716	370,622	
Oncology & HCT hospitalizations, No. (% of all pediatric hospitalizations)						
Oncology (total)	10,123 (3.4)	12,314 (3.5)	20,769 (3.5)	23,401 (4.4)	16,381 (4.4)	<0.001
Hematologic Malignancy	4,292 (1.5)	5,521 (1.6)	9,621 (2.2)	11,530 (2.2)	7,947 (2.1)	<0.001
Solid Malignancy	5,852 (2.0)	6,810 (1.9)	11,183 (1.9)	11,943 (2.2)	8,467 (2.3)	<0.001
HCT	707 (0.2)	1,119 (0.3)	2,086 (0.4)	1,970 (0.4)	1,499 (0.4)	<0.001
Oncology & HCT hospitalizations per 1000 children	1.10	1.11	0.96	1.01	0.95	<0.001
IRR (95% CI)	Reference	1.01 (0.98, 1.03)	0.87 (0.85, 0.90)	0.92 (0.90, 0.94)	0.86 (0.84, 0.88)	
ICU Admission, No. (% of all pediatric hospitalizations)						
Oncology (total)	1,406 (13.9)	1,705 (13.9)	2,988 (14.4)	3,197 (13.7)	2,221 (13.6)	0.22
Hematologic Malignancy	445 (10.4)	599 (10.9)	1,013 (10.5)	1,109 (9.6)	884 (11.1)	0.67
Solid Malignancy	968 (16.5)	1,110 (16.3)	1,982 (17.7)	2,103 (17.6)	1,341 (15.8)	0.83
HCT	169 (23.9)	280 (25.0)	546 (26.2)	462 (23.5)	292 (19.5)	0.002
Oncology & HCT ICU admissions per 1000 children	0.16	0.16	0.15	0.15	0.13	<0.001
IRR (95% CI)	Reference	1.03 (0.96, 1.10)	0.93 (0.88, 0.99)	0.91 (0.86, 0.97)	0.83 (0.78, 0.89)	
Characteristics of oncology & HCT ICU admissions						
Age in years, median (IQR)	4.4 (2.1-7.4)	4.3 (2.3-7.2)	4.3 (2.3-7.3)	4.6 (2.4-7.5)	4.3 (2.4-7.4)	<0.001
Female, No. (%)	676 (46.1)	821 (44.7)	1,474 (45)	1,467 (42.8)	1,065 (44.9)	0.14
Race & ethnicity, No. (%) *						
White, non-Hispanic	697 (67.1)	795 (64.6)	1,784 (63.1)	1,508 (55.6)	1,171 (58.5)	<0.001
Asian or Pacific Islander	40 (3.9)	42 (3.4)	93 (3.3)	163 (6)	100 (5)	
Black, non-Hispanic	113 (10.9)	146 (11.9)	385 (13.6)	346 (12.8)	257 (12.8)	
Hispanic	102 (9.8)	138 (11.2)	350 (12.4)	402 (14.8)	351 (17.5)	
Native American	**	**	18 (0.6)	34 (1.3)	20 (1)	
Another race	82 (7.9)	103 (8.4)	197 (7)	260 (99.5)	104 (5.2)	
Payor, No. (%)						
Public	333 (22.7)	541 (29.5)	1,157 (35.4)	1,389 (40.6)	1,069 (45.3)	<0.001
Private	1,030 (70.3)	1,194 (65)	1,952 (59.7)	1,859 (54.3)	1,118 (47.3)	
Other	102 (7)	101 (5.5)	161 (4.9)	177 (5.2)	175 (7.4)	

*Missing race/ethnicity of 426 encounters in 2001, 605 encounters in 2004, 443 encounters in 2010, 712 encounters in 2016, and 359 encounters in 2019. **Insufficient number to report per HCUP data use agreement. HCT, hematopoietic stem cell transplant; IQR, interquartile range; Mo., month; No., number. Total oncology includes oncology not otherwise specified and HCT includes both oncology and non-oncology transplant admissions. HCT includes non-oncologic indications for transplant. Oncology (total) includes hematologic malignancies, solid malignancies, and malignancies not otherwise specified.

Among all oncology and HCT patients, age did not change substantially over time with a median age of 4.3 years (IQR 2.4-7.4) in 2019 (**Table 1**). The percentage of non-Hispanic White patients decreased from 67.1% in 2001 to 58.5% in 2019, while the percentage of Hispanic patients increased from 9.8% in 2001 to 17.5% in 2019. In contrast, Hispanic patients in the non-oncology ICU population only increased from 12.5% to 16.1%. The use of public insurance among oncology and HCT patients admitted to an ICU doubled from 22.7% in 2001 to 45.5% in 2019 (RR 1.99, 95% CI 1.79-2.21) (**Table 1**).

### Hospital and ICU admission trends

From 2001 to 2019, children with oncologic diagnoses comprised an increasing percentage of hospital admissions, rising from 3.4% in 2001 to 4.4% in 2019, and the percentage of HCT admissions increased from 0.2% in 2001 to 0.4% in 2019. (**Table 1**). ICU admission prevalence among hospitalized patients remained similar for patients with either hematologic or solid malignancies, while it decreased for HCT admissions from a peak of 26.2% in 2010 to 19.5% in 2019 (p=0.002).

In contrast, the population-based rate of combined oncology and HCT admissions decreased from 1.10 to 0.95 hospitalizations per 1000 children per year during that same period (IRR 0.86 [95% CI 0.84-0.88]). The population-based rate of ICU admissions for oncology and HCT patients decreased from 0.16 to 0.13 ICU admissions per 1000 children per year from 2001-2019 (IRR 0.83 [95% CI 0.78-0.89]) (**Table 1**).

### Hospital types

Of 520 hospitals in the dataset that admitted children with oncologic conditions or HCTs across the five sample years, only 75 (14.4%) were dedicated children’s hospitals. Of the 180 hospitals that admitted pediatric oncology and HCT patients to the ICU, only 66 (36.7%) were children’s hospitals. There were 55 ICUs that provided care to >25 oncology or HCT patients in any given year, and of those, 38 (69.1%) were in a children’s hospital.

Patients were increasingly admitted to ICUs in children’s hospitals over the course of the study period, rising from 62.5% to 92.9% of oncology admissions (p<0.001) and from 71.0% to 88.8% of HCT admissions from 2001-2019 (p<0.001) (**Supplementary Table 3**). The prevalence of ICU admission for oncology patients declined from 14.1% in 2001 to 13.3% in 2019 (p=0.003) in children’s hospitals and increased from 13.6% to 14.5% in general hospitals. Among HCT patients, the prevalence of ICU admissions decreased from 33.7% in 2001 to 18.1% in 2019 in children’s hospitals and increased from 14.0% to 40.5% over the same period in general hospitals. Oncology and HCT patients treated in children’s hospitals were of similar age to patients treated in general hospitals but had higher prevalence of non-oncologic and non-hematologic comorbid conditions with higher in-hospital mortality and hospitalization costs (**Supplementary Table 3**).

### Patient clinical characteristics

**Figure 1:**
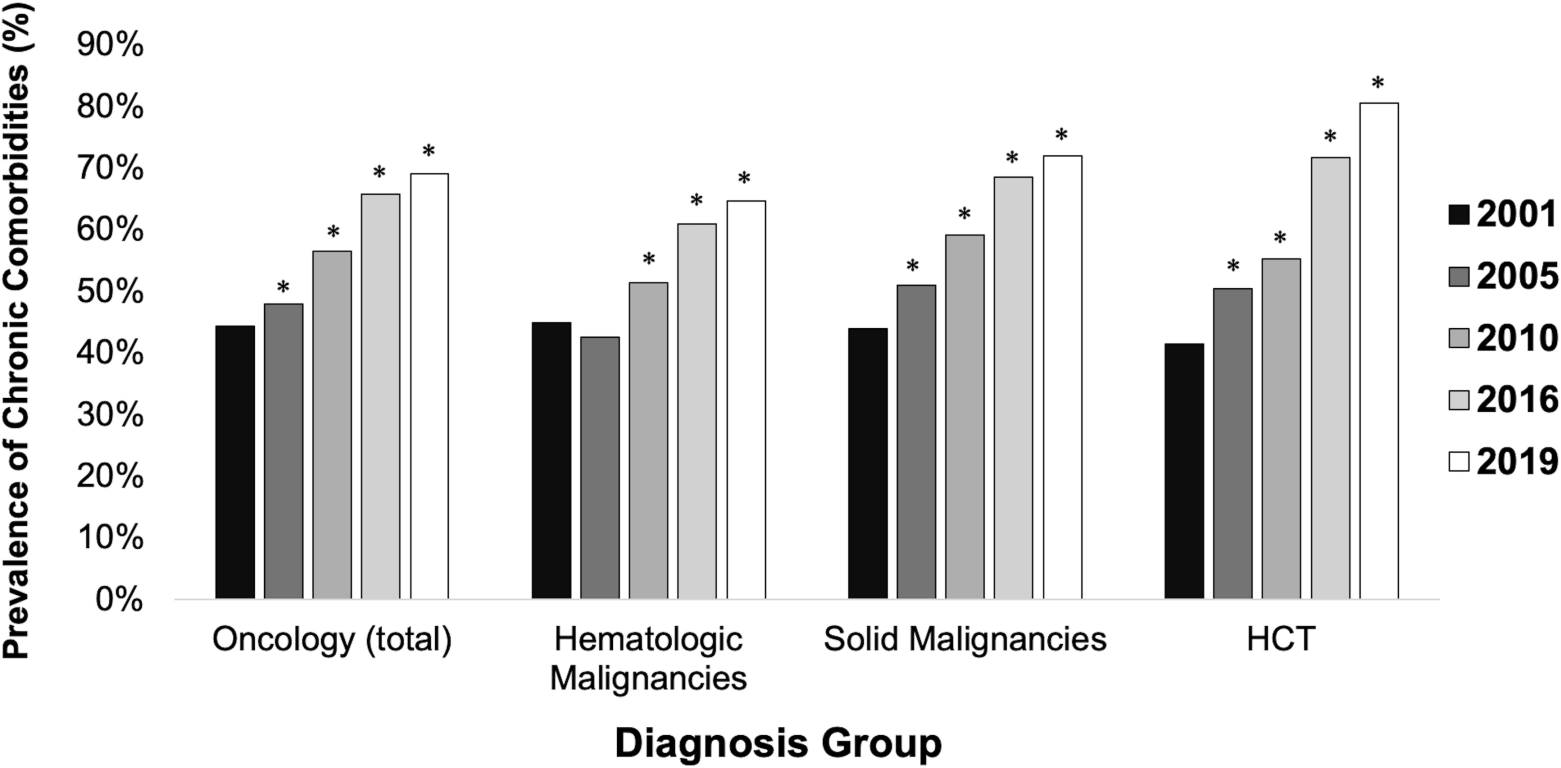
Prevalence of chronic comorbidities among intensive care unit pediatric patients by diagnosis group. * indicates statistically significant relative risk compared to referent group (2001). HCT = hematopoietic stem cell transplant. Comorbidities excluded oncologic and hematologic categories.

The prevalence of chronic non-oncologic and non-hematologic comorbidities among children admitted for an oncologic diagnosis increased from 44.3% in 2001 to 69.1% in 2019 (RR 1.56 [95% CI 1.46-1.66]) (**Figure 1**). Children with HCTs had a greater increase in prevalence of chronic comorbidities from 41.4% to 80.5% (RR 1.94 [95% CI 1.61-2.34]) (**Table 2**). In 2019, the most common types of comorbidities among patients with hematologic malignancies were metabolic (35.5%), renal (21.7%), and cardiovascular (15.4%), while patients with solid malignancies most commonly had neuromuscular comorbidities (44.4%). The most common comorbidities among patients with HCTs were metabolic (33.2%) and gastrointestinal (31.9%). Each group had high rates of technology dependence, including 29.8% of patients with hematologic malignancies, 41.5% of patients with solid malignancies, and 52.1% of HCT patients (**Supplementary Table 4**).

**Table 2:** Clinical traits of pediatric oncology and hematopoietic cell transplant patients admitted to U.S. intensive care units.

Clinical characteristic	2001	2004	2010	2016	2019	p-value
Chronic comorbidities, No. (%)						
Oncology (total)	623 (44.3)	819 (48.0)	1,688 (56.5)	2,104 (65.8)	1,534 (69.1)	
RR (95% CI)	Reference	1.08 (1.00, 1.17)	1.27 (1.19, 1.36)	1.49 (1.39, 1.58)	1.56 (1.46, 1.66)	<0.001
Hematologic Malignancy	200 (44.9)	255 (42.6)	521 (51.4)	675 (60.9)	571 (64.6)	
RR (95% CI)	Reference	0.95 (0.82, 1.09)	1.14 (1.02, 1.29)	1.35 (1.21, 1.52)	1.44 (1.28, 1.61)	<0.001
Solid Malignancy	424 (43.8)	566 (51.0)	1,171 (59.1)	1,441 (68.5)	966 (72.0)	
RR (95% CI)	Reference	1.16 (1.06, 1.28)	1.35 (1.24, 1.46)	1.56 (1.45, 1.69)	1.64 (1.52, 1.78)	<0.001
HCT	70 (41.4)	141 (50.4)	302 (55.3)	331 (71.7)	235 (80.5)	
RR (95% CI)	Reference	1.22 (0.98, 1.51)	1.34 (1.10, 1.62)	1.73 (1.43, 2.09)	1.94 (1.61, 2.34)	<0.001
Any organ failure, No. (%)						
Oncology (total)	515 (36.6)	662 (38.8)	1,552 (51.9)	1,980 (61.9)	1,415 (63.7)	
RR (95% CI)	Reference	1.06 (0.97, 1.16)	1.42 (1.31-1.53)	1.69 (1.57-1.82)	1.74 (1.61-1.88)	<0.001
Hematologic Malignancy	228 (51.2)	293 (48.9)	714 (70.4)	881 (79.4)	679 (76.8)	
RR (95% CI)	Reference	0.95 (0.84, 1.08)	1.37 (1.24, 1.52)	1.55 (1.41, 1.71)	1.50 (1.36 1.65)	<0.001
Solid Malignancy	289 (29.9)	371 (33.4)	843 (42.5)	1,112 (52.9)	740 (55.2)	
RR (95% CI)	Reference	1.12 (0.99, 1.27)	1.42 (1.28, 1.59)	1.77 (1.60, 1.97)	1.85 (1.66, 2.06)	<0.001
HCT	85 (49.7)	149 (53.2)	332 (60.8)	343 (74.2)	203 (69.5)	
RR (95% CI)	Reference	1.07 (0.89, 1.29)	1.22 (1.04, 1.44)	1.49 (1.27, 1.75)	1.40 (1.18, 1.66)	<0.001
MODS, No. (%)						
Oncology (total)	133 (9.5)	198 (11.6)	636 (21.3)	1,000 (31.3)	740 (33.3)	
RR (95% CI)	Reference	1.23 (1.0, 1.51)	2.25 (1.89, 2.68)	3.31 (2.79, 3.92)	3.52 (2.97, 4.18)	<0.001
Hematologic Malignancy	85 (19.1)	112 (18.7)	366 (36.1)	523 (47.2)	432 (48.9)	
RR (95% CI)	Reference	0.98 (0.76, 1.26)	1.89 (1.54, 2.33)	2.47 (2.02, 3.02)	2.56 (2.09, 3.13)	<0.001
Solid Malignancy	50 (5.2)	87 (7.8)	272 (13.7)	484 (23.0)	310 (23.1)	
RR (95% CI)	Reference	1.52 (1.08, 2.13)	2.66 (1.98, 3.56)	4.46 (3.36, 5.90)	4.48 (3.36, 5.96)	<0.001
HCT	21 (12.4)	56 (20.0)	160 (29.3)	190 (41.1)	116 (39.7)	
RR (95% CI)	Reference	1.61 (1.01, 2.56)	2.36 (1.55, 3.59)	3.31 (2.19, 5.01)	3.20 (2.09, 4.89)	<0.001

HCT, hematopoietic stem cell transplant; MODS, Multiple Organ Dysfunction Syndrome; No., number. Comorbidities excluded oncologic or hematologic conditions.

By 2019, 63.7% of oncology patients admitted to an ICU had at least one organ failure, an increase from 36.6% in 2001 (RR 1.74 [95% CI 1.61-1.88]) (**Table 2**). Patients with hematologic malignancies had the highest prevalence of organ failure at 76.8% in 2019, while patients with solid malignancies had the greatest rise over time from 29.9% in 2001 to 55.2% in 2019 (RR 1.85 [95% CI 1.66-2.06]). Prevalence of organ failure among HCT admissions increased from 49.7% to 69.5% (RR 1.40 [95% CI 1.18-1.66]). Neurologic failure was the most common organ failure for all subgroups and was highest among patients with hematologic malignancies (41.7%). Cardiovascular failure was the second most common organ failure among patients with hematologic malignancies, doubling from 18% to 36.5% from 2001-2019, while respiratory failure was the second most common organ failure among patients with solid malignancies or HCTs (**Supplementary Table 5**).

From 2001-2019, the percentage of admissions with MODS increased from 9.5% to 33.3% for oncology patients (RR 3.52, 95% CI 2.97-4.18) and from 12.4% to 39.7% for HCT patients (RR 3.20, 95% CI 2.09-4.89). MODS prevalence was highest among patients with hematologic malignancies at 48.9% in 2019 (**Table 2**). Use of mechanical ventilation did not change over time, while both hematologic malignancy and HCT admissions had increasing prevalence of dialysis use, peaking at 7.1% and 7.9% respectively in 2019. The prevalence of extracorporeal membrane oxygenation support (ECMO)was highest among patients with hematologic malignancies, increasing from 0.3% in 2001 to 1.2% in 2019 (**Supplementary Table 6**). ECMO use ranged from 0% to 1%, depending on the year, in patients with HCT.

### Patient outcomes

The median hospital LOS remained unchanged for all groups between 2001 and 2019, with the longest LOS among patients with hematologic malignancies. Despite stable LOS, hospitalization costs increased in each group. Hematologic malignancy admissions had the highest median cost, reaching $51,895 in 2019, and HCT admissions had the largest increase from $23,622 in 2001 to $40,958 per admission in 2019 (p<0.001) (**Table 3**).

**Table 3:** Outcomes of pediatric oncology and hematopoietic cell transplant patients admitted to U.S. intensive care units.

Clinical Outcome	2001	2004	2010	2016	2019	p-value
Length of stay, Median (IQR)						
Oncology (total)	8 (4-18)	8 (4-16)	8 (4-16)	8 (4-19)	8 (4-18)	0.17
Linear coefficient (95% CI)	Reference	-1.05 (-2.63, 0.52)	-0.54 (-1.96, 0.87)	1.00 (-0.41, 2.40)	1.61 (0.11, 3.10)	
Hematologic Malignancy	12 (5-33)	11 (4-29)	11 (5-29)	14 (6-30)	11 (4-28)	0.15
Linear coefficient (95% CI)	Reference	-3.37 (-6.84, 0.11)	-1.43 (-4.59, 1.73)	1.00 (-2.12, 4.12)	0.11 (-3.11, 3.34)	
Solid Malignancy	7 (4-13)	7 (4-13)	6 (4-12)	6 (4-13)	7 (4-14)	0.23
Linear coefficient (95% CI)	Reference	-0.26 (-1.76, 1.24)	-0.36 (-1.69, 0.98)	0.54 (-0.79, 1.86)	1.23 (-0.21, 2.67)	
HCT	8 (3-24)	8 (4-25)	7 (4-24)	8 (4-21)	6 (4-17)	0.56
Linear coefficient (95% CI)	Reference	-2.45 (-8.83, 3.92)	-1.07 (-4.69, 6.83)	-0.06 (-5.94, 5.83)	-0.38 (-6.71, 5.95)	
Hospitalization cost in 2019 USD, median (IQR)						
Oncology (total)	25662 (13418, 56421)	29268 (14453, 62419)	30631 (16036, 64140)	45519 (23145, 88175)	43848 (21527, 86138)	<0.001
Linear coefficient (95% CI)	Reference	22455 (-7418, 12328)	10171 (1158, 19184)	34992 (25907, 44076)	43138 (33752, 52525)	
Hematologic Malignancy	35193 (12361, 112057)	38445 (13256, 94747)	38738 (16163, 93082)	61846 (28263, 138463)	51895 (21242, 119923)	<0.001
Linear coefficient (95% CI)	Reference	-1870 (-25498, 21757)	8922 (-12843, 30687)	48252 (26346, 70157)	49648 (27701, 71575)	
Solid Malignancy	23966 (13668, 45176)	27022 (14724, 48494)	28661 (15977, 53834)	41070 (22149, 66910)	29917 (21716, 71822)	<0.001
Linear coefficient (95% CI)	Reference	2956 (-5550, 11461)	9021 (1297, 16745)	25756 (17969, 33543)	32716 (24484, 40948)	
HCT	23622 (11839, 92588)	31808 (12338, 89574)	28153 (13581, 99190)	53805 (24087, 126024)	40958 (17644, 89164)	<0.001
Linear coefficient (95% CI)	Reference	-14319 (-59766, 31128)	7581 (-34760, 49925)	41210 (-3496, 85916)	25772 (-19706, 71251)	
Mortality, No. (%)						
Oncology (total)	80 (5.7)	103 (6.0)	122 (4.1)	160 (5.0)	119 (5.4)	0.36
RR (95% CI)	Reference	1.06 (0.80, 1.41)	0.72 (0.54, 0.94)	0.88 (0.68, 1.14)	0.94 (0.71, 1.24)	
Hematologic Malignancy	42 (9.4)	57 (9.5)	73 (7.2)	73 (6.6)	55 (6.2)	0.003
RR (95% CI)	Reference	1.00 (0.69, 1.47)	0.76 (0.53, 1.10)	0.70 (0.48, 1.00)	0.66 (0.45, 0.97)	
Solid Malignancy	40 (4.1)	47 (4.2)	50 (2.5)	88 (4.2)	65 (4.9)	0.29
RR (95% CI)	Reference	1.02 (0.68, 1.55)	0.61 (0.41, 0.92)	1.01 (0.70, 1.46)	1.17 (0.80, 1.72)	
HCT	21 (12.4)	35 (12.5)	43 (7.9)	43 (9.3)	21 (7.2)	0.029
RR (95% CI)	Reference	1.00 (0.61, 1.67)	0.63 (0.39, 1.04)	0.75 (0.46, 1.22)	0.58 (0.33, 1.03)	

HCT, hematopoietic stem cell transplant; IQR, interquartile range; No., number. Costs adjusted to 2019 USD. P-values derived from Cuzick’s test for trend.

Hospital mortality for oncology admissions overall ranged from 4.1-5.7% in each included year, with no change from 2001 to 2019. Mortality declined for admissions with hematologic malignancies from 9.4% in 2001 to 6.2% in 2019 (RR 0.66 [95% CI 0.45-0.97]) while mortality for solid malignancy admissions remained unchanged, ranging from 2.5-4.9% in each year. Mortality for HCT admissions declined from 12.4% in 2001 to 7.2% in 2019 (RR 0.58 [95% CI 0.33-1.03]) (**Table 3**).

### Results by specific diagnoses

Among hematologic malignancies, hospitalized patients with acute lymphoblastic leukemia (ALL) and lymphoma had an increasing ICU admission prevalence, rising from 8.8% to 10.4% of admissions for patients with ALL and from 8.5% to 10.2% of admissions for patients with lymphoma from 2001-2019. Among solid tumors, brain tumors had the largest increase in ICU admission prevalence from 28.9% to 35.3% of admissions from 2001-2019, while the prevalence of ICU admissions for patients with neuroblastomas decreased from 19.4% to 13.0%. Among HCT patients, ICU admission prevalence was higher for patients with non-oncologic HCTs than oncologic HCTs. ICU admission prevalence declined over time for patients with non-oncologic HCTs from 27.4% in 2001 to 22.5% in 2019 and remained similar for oncology-related HCTs (**Supplementary Table 7**).

The prevalence of MODS increased from 2001-2019 across all oncologic diagnoses evaluated, with the highest prevalence of MODS observed among patients with acute myeloid leukemia (AML), with an increase from 24.4% to 63.3%, and oncologic HCTs, with an increase from 13.6% to 44.0% (**Supplementary Figure 1**). MODS prevalence was lowest among patients with nerve tumors (9.5% in 2019) and brain tumors (19.6%).

Despite the increase in MODS, mortality decreased for patients with AML from 14.6% of ICU admissions in 2001 to 8.5% in 2019, while mortality remained similar from 2001-2019 for patients with other oncologic diagnoses including ALL (7.6% to 7.1%), brain tumors (5.1% to 5.0%), and neuroblastoma (5.9% to 3.9%). Among patients with HCTs, mortality was lower for non-oncologic HCT patients than oncologic HCT patients throughout the time period, but with a greater relative decline for oncologic HCT patients from 15.5% in 2001 to 9.2% in 2019 (**Supplementary Table 7**).

## Discussion

In the largest study to date of longitudinal trends in the critically ill pediatric oncology and HCT population in the U.S., we found that oncology and HCT patients requiring ICU care are becoming increasingly medically complex with rising prevalence of MODS. Organ dysfunction was most prevalent among patients with hematologic malignancies, particularly AML. Despite this, mortality remained similar or improved across all oncology and HCT subtypes. By using a large, population-based dataset including patients from 21 states and 520 general and children’s hospitals, we have for the first time provided an assessment of ICU admission trends and outcomes across the range of facilities caring for children with oncologic conditions and HCTs. We have augmented previous studies by examining mortality and morbidity trends in unique oncologic and HCT subgroups. With this new information, clinicians can target specific patient populations for further research and intervention.

Previous studies have found an increase in the ICU admission rate for oncology patients (4, 5, 24) yet our study did not find a universal trend across groups, with ICU admission prevalence among hospitalized patients rising for patients with ALL, lymphoma, and brain tumors but remaining similar or decreasing in other groups. These trends may be in part due to novel therapeutics, such as chimeric antigen receptor T cells (CAR-T), being more readily available for certain types of diagnoses and not others. Although, generally well-tolerated, CAR-T therapy has known side effects such as Cytokine Release Syndrome (CRS) or Immune effector cell-Associated Neurotoxicity Syndrome (ICANS) that frequently result in ICU-level of care (25–27). Other immunotherapies have expanded in their application, as is the case of bispecific T cell engagers like blinatumomab. These therapies, used both in primary and relapsed leukemias, can be accompanied fever, hypotension, or encephalopathy (28). Declines in ICU admission prevalence over time among patients with neuroblastoma and both oncologic and non-oncologic HCTs may be due to changes in treatment regimens and improved management of therapeutic toxicities (29). To our knowledge, no other study investigated ICU admissions by both broad and specific oncologic diagnoses; by examining both of these categories, our study provides insight into which diagnoses may be driving overall trends.

We found that pediatric oncology and HCT patients are being admitted to the ICU with greater underlying and acute complexity: a higher prevalence of chronic comorbid conditions, at least one organ failure, and MODS. Oncology patients frequently require interventions such as mechanical ventilation, inotropic support, or central vascular access due to acute illness severity (8), and together with HCT patients have higher illness severity scores and mortality than the general pediatric ICU population (9, 13, 17, 30). We found that neurologic failure was the most frequent organ failure in all groups. Neurologic complications in pediatric oncology patients are common, and there are many potential etiologies including effects of chemotherapy, radiation, infection, procedures, surgery, and the neoplasm itself (31). The diversity of etiologies may partially explain why patients experienced neurologic failure at higher rates than other organ systems. Another common organ failure was cardiovascular compromise. Sepsis may play a role in this increased risk of cardiovascular failure, but our dataset lacks the granular clinical data necessary to make an association between the two. This increase in cardiovascular failure may be multifactorial and related to cardiovascular risk factors, treatment side effects, or an increase in chronic cardiovascular comorbidities. Similarly, the increased prevalence of chronic comorbidities in patients may predispose them to the increased observed prevalence of organ failure and MODS. Patients with relapsed leukemic disease also may experience new baseline organ dysfunction as a consequence of their disease and treatment requirements (32). Their disease may be salvageable but necessitate more frequent ICU care. Our findings of increased organ failures over time suggest that illness severity in these populations continues to rise and that clinicians will be increasingly managing multi-organ failure in these vulnerable patients. In the context of existing immense variability in resource use (33), hospitals and ICUs may need to dedicate additional ICU staff and resources to care for children with oncologic conditions and HCTs given the escalating proportion of admissions with severe illness and high resource needs.

Despite changes in illness severity, we did not find any change in LOS across the groups. We did, however, identify an increase in hospital costs, which may be related to increased illness severity and ICU interventions and costs of new cancer therapies. We noted a trend of increased ECMO use in oncologic diagnoses, which is consistent with recent studies (34, 35), and may contribute to the rising costs. Access to specialized cancer therapies and associated clinical trials may also be contributing to the increasing prevalence of oncology and HCT care occurring in specialized pediatric hospitals. Increasing regionalization of care for critically ill pediatric oncology and HCT patients may contribute to improved outcomes (36), though may also contribute to delays in care for patients living in geographically disperse areas (37). Some evidence suggests that increased geographical distance to treatment centers portends worse mortality trends in ALL (38). However, one study conducted in rural Virginia found comparable survival outcomes in adults with AML (39). While further studies are needed to determine the extent of travel that patients require to receive specialized care and how it impacts severity of illness and outcomes, investment in supplementary efforts such as telehealth, education, and collaboration with local healthcare providers may reduce undesirable outcomes. Importantly, we found that many general hospitals continue to admit children with cancer and HCTs to both the inpatient ward and ICUs, suggesting the need to include both children’s and non-children’s hospitals in efforts to improve processes of care and outcomes for pediatric oncology and HCT populations.

There were several limitations in our study. First, the included states varied each year, and only four states were included in all 5 years of data analyzed. Second, all counts refer to admissions rather than patients so we cannot determine the extent to which encounters were representative of individual patients or readmissions of the same patient. Third, we excluded patients 18 years or older despite many pediatric hospitals treating young adult patients with oncologic diagnoses, and exclusion of these patients limits our ability to understand their contribution to trends and findings. Fourth, diagnoses, comorbid conditions, and organ failures were determined by ICD codes, and changes in prevalence may reflect changes between ICD-9 and ICD-10 coding, which has imperfect sensitivity and specificity. Changes in coding practices over time may have particularly influenced the frequency with which comorbid conditions and organ failures were recorded. Finally, we were unable to identify the timing of events such as development of organ failures or mortality and thus cannot determine whether they occurred during the ICU stay.

## Conclusions

Pediatric oncology and HCT patients are increasingly medically complex, with increasing prevalence of baseline comorbid conditions and increasing illness severity if admitted to an ICU. Despite this, mortality has improved for critically ill oncology and HCT patients. While care is becoming increasingly regionalized to children’s hospitals, many general hospitals continue to admit pediatric oncology and HCT patients, and ICU admission prevalence varies widely based on the type of hospital in which they receive care. These facilities will increasingly need to devote ICU resources to care for these children, especially with the ongoing development of novel immunotherapies, and further support for both children’s and general hospitals may be needed to continue to improve outcomes.

## Data Availability

The data analyzed in this study is subject to the following licenses/restrictions: Cost, Data Use Agreement. Requests to access these datasets should be directed to https://hcup-us.ahrq.gov/sidoverview.jsp.
