# Supplementary material for: The epidemiology of pediatric oncology and hematopoietic cell transplant admissions to U.S. intensive care units from 2001-2019
Source: Front Oncol. 2024 Dec 3;14:1501977. doi: 10.3389/fonc.2024.1501977 (PMC11653354; doi:10.3389/fonc.2024.1501977)
Supplement: Supplementary file 1 [file DataSheet1.pdf]

Online-only Supplement

**eTable 1:** States that contributed to the Healthcare Cost and Utilization Project dataset by year	Page 2
**eTable 2:** International Classification of Disease Clinical Modification -9 and -10 codes for classification of disease	Pages 3-4
**eTable 3:** Patient characteristics and outcomes by hospital type	Pages 5-6
**eTable 4:** Chronic comorbid conditions among pediatric oncology and hematopoietic cell transplant patients admitted to U.S. intensive care units from 2001-2019	Pages 7-8
**eTable 5:** Organ failure type by diagnosis category by year	Page 9
**eTable 6:** Intensive care unit resource use by diagnosis group by year	Page 10
**eTable 7:** Clinical characteristics and outcomes of individual cancer diagnosis types	Pages 11-12
**eFigure 1:** Prevalence of multiorgan dysfunction syndrome among intensive care unit pediatric patients by diagnosis	Page 13

eTable 1: States that contributed to the Healthcare Cost and Utilization Project dataset by year

2001	2005	2010	2016	2019
		Arkansas	Arkansas	Arkansas
			Delaware	Delaware
		Georgia	Georgia	Georgia
		Iowa	Iowa	Iowa
Kentucky	Kentucky	Kentucky	Kentucky	Kentucky
Massachusetts	Massachusetts	Massachusetts	Massachusetts	Massachusetts
		Maryland	Maryland	Maryland
Maine				
		Minnesota	Minnesota	Minnesota
		Mississippi	Mississippi	
	North Carolina	North Carolina	North Carolina	North Carolina
Nebraska	Nebraska	Nebraska	Nebraska	Nebraska
		New Jersey	New Jersey	New Jersey
			New Mexico	New Mexico
	Nevada		Nevada	Nevada
New York	New York	New York	New York	New York
		Oregon	Oregon	Oregon
			Utah	
Washington	Washington	Washington	Washington	Washington
		Wisconsin	Wisconsin	Wisconsin
West Virginia	West Virginia	West Virginia	West Virginia	West Virginia

eTable 2: International Classification of Disease Clinical Modification -9 and -10 codes for classification of disease

**ICD-CM-9**		
**Immunocompromised Category**	**Subclassification**	**Code**
Hematologic Malignancy	Acute Lymphoid Leukemia	204.0, V10.61
	Acute Myeloid Leukemia	205.0, V10.62
	Chronic Myeloid Leukemia	205.1
	Hodgkins Lymphoma	201.x, V10.72
	Non-Hodgkins Lymphoma	200.x (except for 200.2), 202.x
	Burkitt Lymphoma	200.2
	Myelodysplastic Syndrome	238.7
	Other Hematologic Malignancy	203.x-208.x (except for 204.0 and 205.0), V10.63
Solid Malignancy	Brain Tumor	191.x, 192.x, 194.4, 239.6, V10.85
	Osteosarcoma	170.x, V10.81
	Soft Tissue Sarcoma	171.x, 239.2
	Neuroblastoma	194.x
	Nephroblastoma	189.x, V10.52
	Hepatoblastoma	155.x, V10.07
	Other Solid Malignancy	140.x-165.x (except for 155.x), 172.x-190.x, 193.x-199.x (except for 194.4), 239.x (except for 239.2), V10.29, V10.41, V10.43, V10.47
Congenital Immunodeficiency	Severe Combined Immunodeficiency	279.2
	Non-SCID Immunodeficiency	42.x, 279.0, 279.1, V08
Aplastic Anemia		284.x (except for 284.01 and 284.81)
Hemophagocytic Lymphohistiocytosis		288.4
Transplant	Bone marrow transplant	V42.81
**ICD-CM-10**		
**Immunocompromised Category**	**Subclassification**	**Code**
Hematologic Malignancy	Acute Lymphoid Leukemia	C91
	Myeloid Leukemia	C92
	Acute Myeloid Leukemia with multilineage dysplasia	C92.A
	Chronic Myeloid Leukemia (BCR/ABL +)	C92.1
	Atypical Chronic Myeloid Leukemia (BCR/ABL -)	C92.2
	Hodgkin Lymphoma	C81
	Follicular Lymphoma	C82
	Non-follicular Lymphoma (including Burkitt)	C83
	Other specified and unspecified types of non-Hodgkin Lymphoma	C85
	Myelodysplastic Syndrome	D46
Solid Malignancy	Brain Tumor	C71
	Spinal cord and cranial nerve tumor	C72

Immunocompromised Category	Subclassification	Code
	Osteosarcoma (limbs)	C40
	Osteosarcoma (unspecified sites)	C41
	Peripheral nerves and autonomic nervous system tumor	C47
	Retroperitoneum and peritoneum tumor	C48
	Connective and soft tissue tumor	C49
	Neuroblastoma (adrenal glands)	C74
	Nephroblastoma	C64, C65
	Hepatoblastoma	C22.2
	Digestive organ tumors	C15-C26
Congenital Immunodeficiency	Severe Combined Immunodeficiency	D81.0, D81.1, D81.2
	Non-SCID Immunodeficiency	D82
Aplastic Anemia	Aplastic Anemia, unspecified	D61.9
Hemophagocytic Lymphohistiocytosis		D76.1
Transplant	History of bone marrow transplant	Z94.81, Z94.84

[a] Where three-digit and four-digit codes are listed, all associated subcodes are also included unless otherwise specified. ICD-CM-9 and -10 = International Classification of Disease Clinical Modification

eTable 3: Patient characteristics and outcomes by hospital type

Patient characteristic	2001	2005	2010	2016	2019	p-value
**Children's Hospital Admissions**, No. (%)						
Oncology (total)	878 (62.5)	1239 (74.5)	2431 (81.4)	2777 (88.0)	1981 (92.9)	<0.001
RR (95% CI)	Reference	1.19 (1.14, 1.25)	1.30 (1.25, 1.36)	1.41 (1.35, 1.47)	1.49 (1.42, 1.55)	
Hematologic Malignancies	288 (64.7)	439 (74.4)	841 (83.0)	964 (88.9)	764 (92.1)	<0.001
RR (95% CI)	Reference	1.15 (1.06, 1.25)	1.28 (1.19, 1.38)	1.37 (1.28, 1.48)	1.42 (1.32, 1.53)	
Solid Malignancies	592 (61.2)	802 (74.5)	1595 (80.5)	1825 (87.5)	1221 (93.5)	<0.001
RR (95% CI)	Reference	1.22 (1.15, 1.30)	1.32 (1.25, 1.39)	1.43 (1.36, 1.51)	1.53 (1.45, 1.61)	
HCT	120 (71.0)	226 (81.9)	489 (89.6)	408 (89.1)	254 (88.8)	<0.001
RR (95% CI)	Reference	1.15 (1.03, 1.29)	1.26 (1.14, 1.39)	1.25 (1.13, 1.39)	1.25 (1.13, 1.39)	
**ICU Admission**, No. (% of hospitalizations)						
Oncology (total), Children's hospital	878 (14.1)	1239 (14.5)	2431 (14.4)	2777 (13.5)	1981 (13.3)	0.003
RR (95% CI)	Reference	1.03 (0.95, 1.11)	1.02 (0.95, 1.10)	0.96 (0.90, 1.03)	0.94 (0.88, 1.02)	
Oncology (total), General hospital	528 (13.6)	424 (12.7)	557 (14.6)	378 (15)	151 (14.5)	0.025
RR (95% CI)	Reference	0.93 (0.83, 1.05)	1.07 (0.96, 1.19)	1.10 (0.97, 1.24)	1.06 (0.90, 1.25)	
HCT, Children's hospital	120 (33.7)	226 (31.8)	489 (27.5)	408 (23.6)	254 (18.1)	<0.001
RR (95% CI)	Reference	0.94 (0.79, 1.13)	0.82 (0.69, 0.96)	0.7 (0.59, 0.83)	0.54 (0.44, 0.65)	
HCT, General hospital	49 (14)	50 (12.7)	57 (18.6)	50 (21.9)	32 (40.5)	<0.001
RR (95% CI)	Reference	0.91 (0.63, 1.31)	1.33 (0.94, 1.89)	1.57 (1.10, 2.25)	2.9 (2, 4.2)	
**Age of ICU admissions**, Median years (IQR)						
Oncology & HCT, Children's hospital	7 (3, 12)	7 (3, 13)	7 (3, 12)	8 (3, 13)	8 (3, 13)	<0.001
RR (95% CI)	Reference	1.01 (0.95, 1.07)	1.02 (0.97, 1.07)	1.07 (1.02, 1.13)	1.07 (1.01, 1.13)	
Oncology & HCT, General hospital	7 (3, 13)	7 (3, 13)	7 (2, 13)	8 (3, 13)	8.5 (5, 14)	0.144
RR (95% CI)	Reference	1.02 (0.94, 1.11)	0.97 (0.90, 1.06)	1.03 (0.95, 1.12)	1.13 (1.02, 1.25)	
**Chronic comorbidities**, No (%)						
Oncology & HCT, Children's hospital	418 (45.3)	660 (49.2)	1,583 (58.7)	2,031 (68.1)	1,519 (71.7)	<0.001
RR (95% CI)	Reference	1.09 (0.99, 1.19)	1.30 (1.20, 1.4)	1.50 (1.39, 1.62)	1.58 (1.47, 1.71)	
Oncology & HCT, General hospital	234 (43.2)	218 (48.4)	276 (47.8)	236 (58.1)	105 (64.8)	<0.001
RR (95% CI)	Reference	1.12 (0.98, 1.29)	1.11 (0.97, 1.26)	1.34 (1.19, 1.53)	1.5 (1.29, 1.74)	

Patient characteristic (continued)	2001	2005	2010	2016	2019	p-value
**Hospitalization cost in 2019 USD,** Median (IQR)						
Oncology & HCT, Children's hospital	27067 (13308, 63152)	30539 (14596, 68142)	31818 (16187, 68805)	45993 (23292, 92860)	44859 (23016, 90092)	
Oncology & HCT, General hospital	22762 (13520, 45282)	26837 (12256, 49739)	24500 (12668, 42773)	43451 (21192, 70524)	32535 (15609, 66139)	
**In-hospital mortality**, No (%)						
Oncology (total), Children's hospital	47 (5.4)	72 (5.8)	108 (4.4)	136 (4.9)	112 (5.7)	0.94
RR (95% CI)	Reference	1.08 (0.76, 1.55)	0.83 (0.59, 1.16)	0.91 (0.66, 1.26)	1.06 (0.76, 1.47)	
Oncology (total), General hospital	33 (6.3)	28 (6.6)	14 (2.5)	23 (6.1)	7 (4.6)	0.22
RR (95% CI)	Reference	1.06 (0.65, 1.72)	0.40 (0.22, 0.74)	0.97 (0.58, 1.63)	0.74 (0.33, 1.64)	
HCT, Children's hospital	13 (10.8)	25 (11.1)	42 (8.6)	39 (9.6)	21 (8.3)	0.36
RR (95% CI)	Reference	1.02 (0.54, 1.92)	0.79 (0.44, 1.43)	0.88 (0.49, 1.60)	0.76 (0.40, 1.47)	
HCT, General hospital	8 (16.3)	10 (20)	1 (1.8)	4 (8)	0 (0)	0.002
RR (95% CI)	Reference	1.22 (0.53, 2.85)	0.11 (0.01, 0.83)	0.49 (0.16, 1.53)	-	

HCT = hematopoietic stem cell transplant, IQR = interquartile range, No. = number, RR = relative risk, - = unable to calculate.

eTable 4: Chronic comorbid conditions among pediatric oncology and hematopoietic cell transplant patients admitted to U.S. intensive care units from 2001-2019

**Chronic Comorbidity**, No. (%)	**2001**	**2004**	**2010**	**2016**	**2019**
**Oncology (total)**					
Cardiovascular	168 (12)	240 (14.1)	325 (10.9)	443 (13.9)	281 (12.7)
Genetic	30 (2.1)	37 (2.2)	114 (3.8)	116 (3.6)	107 (4.8)
Gastrointestinal	69 (4.9)	110 (6.5)	235 (7.9)	371 (11.6)	304 (13.7)
Metabolic	75 (5.3)	100 (5.9)	469 (15.7)	741 (23.2)	566 (25.5)
Neuromuscular	255 (18.1)	326 (19.1)	725 (24.3)	944 (29.5)	655 (29.5)
Renal	177 (12.6)	233 (13.7)	458 (15.3)	435 (13.6)	412 (18.6)
Respiratory	21 (1.5)	31 (1.8)	59 (2)	116 (3.6)	89 (4)
Technology dependence[a]	374 (26.6)	525 (30.8)	829 (27.7)	1078 (33.7)	817 (36.8)
**Hematologic Malignancy**					
Cardiovascular	95 (21.4)	123 (20.5)	145 (14.3)	199 (17.9)	136 (15.4)
Genetic	18 (4.0)	20 (3.3)	60 (5.9)	59 (5.3)	72 (8.1)
Gastrointestinal	19 (4.3)	28 (4.7)	71 (7.0)	100 (9.0)	101 (11.4)
Metabolic	31 (7.0)	36 (6.0)	211 (20.8)	393 (35.4)	314 (35.5)
Neuromuscular	18 (4.0)	26 (4.3)	58 (5.7)	79 (7.1)	60 (6.8)
Renal	78 (17.5)	94 (15.7)	180 (17.8)	158 (14.3)	192 (21.7)
Respiratory	9 (2.0)	4 (0.7)	11 (1.1)	21 (1.9)	27 (3.1)
Technology dependence[a]	139 (31.2)	179 (29.9)	231 (22.8)	273 (24.6)	263 (29.8)
**Solid Malignancy**					
Cardiovascular	73 (7.5)	117 (10.5)	180 (9.1)	246 (11.7)	145 (10.8)
Genetic	12 (1.2)	17 (1.5)	54 (2.7)	57 (2.7)	35 (2.6)
Gastrointestinal	50 (5.2)	83 (7.5)	164 (8.3)	273 (13.0)	204 (15.2)
Metabolic	45 (4.7)	64 (5.8)	260 (13.1)	352 (16.7)	253 (18.9)
Neuromuscular	237 (24.5)	300 (27.0)	668 (33.7)	870 (41.4)	596 (44.4)
Renal	100 (10.3)	140 (12.6)	281 (14.2)	283 (13.5)	222 (16.6)
Respiratory	12 (1.2)	27 (2.4)	48 (2.4)	95 (4.5)	62 (4.6)
Technology dependence[a]	236 (24.4)	348 (31.4)	602 (30.4)	809 (38.5)	556 (41.5)
**HCT**					
Cardiovascular	34 (20.1)	69 (24.6)	70 (12.8)	75 (16.2)	33 (11.3)
Genetic	4 (2.4)	5 (1.8)	33 (6)	33 (7.1)	14 (4.8)
Gastrointestinal	13 (7.7)	32 (11.4)	85 (15.6)	109 (23.6)	93 (31.9)
Metabolic	17 (10.1)	37 (13.2)	108 (19.8)	157 (34)	97 (33.2)
Neuromuscular	9 (5.3)	31 (11.1)	71 (13)	75 (16.2)	49 (16.8)

**Chronic Comorbidity**, No. (%)	**2001**	**2004**	**2010**	**2016**	**2019**
**HCT (continued)**					
Renal	15 (8.9)	30 (10.7)	64 (11.7)	49 (10.6)	75 (25.7)
Respiratory	6 (3.6)	12 (4.3)	20 (3.7)	15 (3.3)	11 (3.8)
Technology dependence[a]	46 (27.2)	94 (33.6)	148 (27.1)	200 (43.3)	152 52.1)

HCT = hematopoietic stem cell transplant, No. = number. [a] Technology dependence defined according to Pediatric Complex Chronic Conditions and includes devices such as tracheostomies, gastrostomies, colostomies, ventricular shunts, dialysis access, and pacemakers.

eTable 5: Organ failure type by diagnosis category by year

**Organ failure**, No. (%)	**2001**	**2004**	**2010**	**2016**	**2019**
**Oncology (total)**					
Cardiovascular	128 (9.1)	194 (11.4)	520 (17.4)	781 (24.4)	565 (25.4)
Hepatic	7 (0.5)	17 (1.0)	32 (1.1)	51 (1.6)	51 (2.3)
Neuromuscular	247 (17.6)	299 (17.5)	887 (29.7)	1209 (37.8)	831 (37.4)
Renal	56 (4.0)	72 (4.2)	147 (4.9)	306 (9.6)	256 (11.5)
Respiratory	163 (11.6)	207 (12.1)	517 (17.3)	692 (21.7)	584 (26.3)
**Hematologic Malignancy**					
Cardiovascular	80 (18.0)	108 (18.0)	284 (28.0)	407 (36.7)	323 (36.5)
Hepatic	4 (0.9)	11 (1.8)	22 (2.2)	29 (2.6)	32 (3.6)
Neuromuscular	89 (20.0)	89 (14.9)	394 (38.9)	503 (45.4)	369 (41.7)
Renal	43 (9.7)	50 (8.4)	107 (10.6)	211 (19.0)	181 (20.5)
Respiratory	80 (18.0)	105 (17.5)	228 (22.5)	301 (27.1)	295 (33.4)
**Solid Malignancy**					
Cardiovascular	49 (5.1)	86 (7.8)	237 (12.0)	380 (18.1)	243 (18.1)
Hepatic	3 (0.3)	7 (0.6)	10 (0.5)	22 (1.1)	19 (1.4)
Neuromuscular	158 (16.3)	210 (18.9)	493 (24.9)	714 (34.0)	464 (34.6)
Renal	14 (1.5)	24 (2.2)	42 (2.1)	99 (4.7)	76 (5.7)
Respiratory	85 (8.8)	103 (9.3)	292 (14.7)	395 (18.8)	291 (21.7)
**HCT**					
Cardiovascular	19 (11.2)	53 (18.9)	136 (24.9)	160 (34.6)	88 (30.1)
Hepatic	2 (1.2)	3 (1.1)	8 (1.5)	8 (1.7)	7 (2.4)
Neuromuscular	41 (24.3)	62 (22.1)	133 (24.4)	173 (37.5)	98 (33.6)
Renal	14 (8.3)	18 (6.4)	66 (12.1)	77 (16.7)	59 (20.2)
Respiratory	28 (16.6)	56 (20.0)	137 (25.1)	137 (29.7)	91 (31.2)

HCT = hematopoietic stem cell transplant, No. = number.

eTable 6: Intensive care unit resource use by diagnosis group by year

Device	2001	2004	2010	2016	2019
**Mechanical Ventilation**					
Oncology (total)	168 (12.0)	196 (11.5)	393 (13.2)	414 (13.0)	279 (12.6)
RR (95% CI)	Reference	0.96 (0.79-1.17)	1.10 (0.93-1.30)	1.08 (0.92-1.28)	1.05 (0.88-1.26)
Hematologic Malignancy	69 (15.5)	81 (13.5)	143 (14.1)	175 (15.8)	119 (13.5)
RR (95% CI)	Reference	0.87 (0.65-1.17)	0.91 (0.70-1.19)	1.02 (0.79-1.31)	0.87 (0.66-1.14)
Solid Malignancy	102 (10.5)	115 (10.4)	251 (12.7)	241 (11.5)	162 (12.1)
RR (95% CI)	Reference	0.98 (0.76-1.27)	1.20 (0.97-1.49)	1.09 (0.87-1.35)	1.15 (0.91-1.45)
HCT	22 (13.0)	39 (13.9)	78 (14.3)	75 (16.2)	38 (13.0)
RR (95% CI)	Reference	1.07 (0.66-1.74)	1.10 (0.71-1.71)	1.25 (0.80-1.94)	1.00 (0.61-1.63)
**Dialysis**					
Oncology (total)	16 (1.1)	24 (1.4)	42 (1.4)	47 (1.54)	83 (3.76)
RR (95% CI)	Reference	1.24 (0.66-2.32)	1.24 (0.70-2.19)	1.29 (0.74-2.27)	3.28 (1.93-5.58)
Hematologic Malignancy	10 (2.3)	17 (2.8)	33 (3.3)	38 (3.4)	63 (7.1)
RR (95% CI)	Reference	1.26 (0.58-2.73)	1.45 (0.72-2.92)	1.52 (0.77-3.03)	3.17 (1.64-6.12)
Solid Malignancy	6 (0.6)	8 (0.7)	9 (0.5)	11 (0.5)	20 (1.5)
RR (95% CI)	Reference	1.16 (0.40-3.34)	0.73 (0.26-2.05)	0.84 (0.31-2.28)	2.41 (0.97-5.97)
HCT	5 (3.0)	8 (2.9)	21 (3.9)	9 (2.0)	23 (7.9)
RR (95% CI)	Reference	0.97 (0.32-2.9)	1.30 (0.50-3.40)	0.66 (0.22-1.94)	2.67 (1.03-6.87)
**ECMO**					
Oncology (total)	4 (0.3)	2 (0.1)	6 (0.2)	9 (0.3)	16 (0.7)
RR (95% CI)	Reference	0.41 (0.08-2.25)	0.71 (0.20-2.50)	1.0 (0.31-3.21)	2.53 (0.85-7.56)
Hematologic Malignancy	1 (0.2)	2 (0.3)	5 (0.5)	8 (0.7)	11 (1.2)
RR (95% CI)	Reference	1.49 (0.14-16.3)	2.20 (0.26-18.8)	3.21 (0.40-25.6)	5.53 (0.72-42.8)
Solid Malignancy	3 (0.3)	0 (0)	1 (0.1)	1 (0.1)	5 (0.4)
RR (95% CI)	Reference	-	-	-	-
HCT	1 (0.6)	0 (0)	1 (0.2)	1 (0.2)	3 (1.0)
RR (95% CI)	Reference	-	-	-	-

ECMO = extracorporeal membrane oxygenation, HCT = hematopoietic stem cell transplant, No. = number. - = unable to calculate

eTable 7: Clinical characteristics and outcomes of individual cancer diagnosis types

Diagnosis	2001	2004	2010	2016	2019
**ICU admission prevalence**, No. (% of hospitalizations)					
Hematologic Malignancy					
ALL	262 (8.8)	341 (9.2)	622 (8.9)	672 (8.8)	566 (10.4)
AML	123 (19.0)	159 (16.7)	283 (19.1)	216 (17.6)	129 (17.3)
Lymphoma	51 (8.5)	95 (12.1)	104 (10.0)	217 (8.7)	164 (10.2)
Other	10 (14.1)	13 (12.8)	18 (10.3)	43 (13.3)	48 (18.8)
Solid Malignancy					
Brain	476 (28.9)	581 (32.4)	993 (33.3)	1040 (33.8)	662 (35.3)
Neuroblastoma	219 (19.4)	215 (17.8)	380 (16.5)	326 (18.2)	182 (13.0)
Hepatoblastoma	50 (19.5)	55 (13.6)	117 (19.5)	103 (17.0)	76 (15.9)
Nephroblastoma	65 (17.5)	77 (15.1)	135 (17.4)	134 (15.0)	111 (18.1)
Bone	101 (6.4)	125 (6.3)	205 (6.6)	210 (6.0)	120 (4.6)
Muscle	57 (6.7)	58 (6.8)	141 (10.1)	81 (7.7)	63 (7.5)
Nerve	22 (33.7)	21 (45.7)	45 (60.8)	99 (25.9)	74 (26.5)
Other	61 (6.5)	65 (6.5)	155 (9.5)	466 (18.5)	253 (13.7)
HCT					
Oncology	110 (22.4)	149 (22.5)	260 (23.8)	226 (22.8)	141 (17.0)
Non-oncology	59 (27.4)	131 (28.7)	286 (28.7)	236 (24.1)	151 (22.5)
**MODS**, No. (%)					
Hematologic Malignancy					
ALL	45 (17.2)	65 (19.1)	211 (33.9)	308 (45.8)	268 (47.4)
AML	30 (24.4)	38 (23.9)	124 (43.8)	127 (58.8)	83 (63.3)
Lymphoma	8 (15.7)	11 (11.6)	34 (32.7)	87 (40.1)	67 (40.9)
Other	2 (20.0)	1 (7.7)	4 (22.2)	25 (58.1)	27 (56.3)
Solid Malignancy					
Brain	22 (4.6)	47 (8.1)	104 (10.5)	164 (15.8)	130 (19.6)
Neuroblastoma	17 (7.8)	22 (10.2)	62 (16.3)	130 (39.9)	56 (30.8)
Hepatoblastoma	0 (0)	6 (10.9)	29 (24.8)	32 (31.1)	32 (42.1)
Nephroblastoma	1 (1.5)	2 (2.6)	17 (12.6)	28 (20.9)	26 (23.4)
Bone	2 (2.0)	6 (4.8)	30 (14.6)	68 (32.4)	32 (26.7)
Muscle	8 (14.0)	3 (5.2)	25 (17.7)	27 (33.3)	22 (34.9)
Nerve	0 (0)	0 (0)	6 (13.3)	24 (24.2)	7 (9.5)
Other	9 (14.8)	4 (6.2)	30 (19.4)	148 (31.8)	67 (26.5)
HCT					
Oncology	15 (13.6)	33 (22.2)	94 (36.2)	111 (49.1)	62 (44.0)
Non-oncology	6 (10.2)	23 (17.6)	66 (23.1)	79 (33.5)	54 (35.8)

Diagnosis (continued)	2001	2004	2010	2016	2019
**In-Hospital Mortality**, No. (%)					
Hematologic Malignancy					
ALL	20 (7.6)	30 (8.8)	34 (5.5)	36 (5.4)	40 (7.1)
AML	18 (14.6)	21 (13.2)	38 (13.4)	22 (10.2)	11 (8.5)
Lymphoma	3 (5.9)	7 (7.4)	4 (3.9)	15 (6.9)	4 (2.4)
Other	1 (10.0)	1 (7.7)	1 (5.6)	4 (9.3)	1 (2.1)
Solid Malignancy					
Brain	24 (5.1)	26 (4.5)	28 (2.8)	39 (3.8)	33 (5.0)
Neuroblastoma	13 (5.9)	7 (3.3)	4 (1.1)	12 (3.7)	7 (3.9)
Hepatoblastoma	1 (2.0)	4 (7.3)	6 (5.1)	4 (3.9)	0 (0)
Nephroblastoma	1 (1.5)	0 (0)	1 (0.7)	5 (3.7)	2 (1.8)
Bone	1 (1.0)	5 (4.0)	7 (3.4)	9 (4.3)	9 (7.5)
Muscle	3 (5.3)	5 (8.6)	3 (2.1)	3 (3.7)	9 (14.3)
Nerve	0 (0)	1 (4.8)	2 (4.4)	5 (5.1)	4 (5.4)
Other	3 (4.9)	6 (9.2)	4 (2.6)	24 (5.2)	9 (3.6)
HCT					
Oncology	17 (15.5)	21 (14.1)	28 (10.8)	21 (9.3)	13 (9.2)
Non-oncology	4 (6.8)	14 (10.7)	15 (5.3)	22 (9.3)	8 (5.3)

HCT = hematopoietic stem cell transplant, ICU = intensive care unit, MODS = Multiple Organ Dysfunction Syndrome, No. = number.

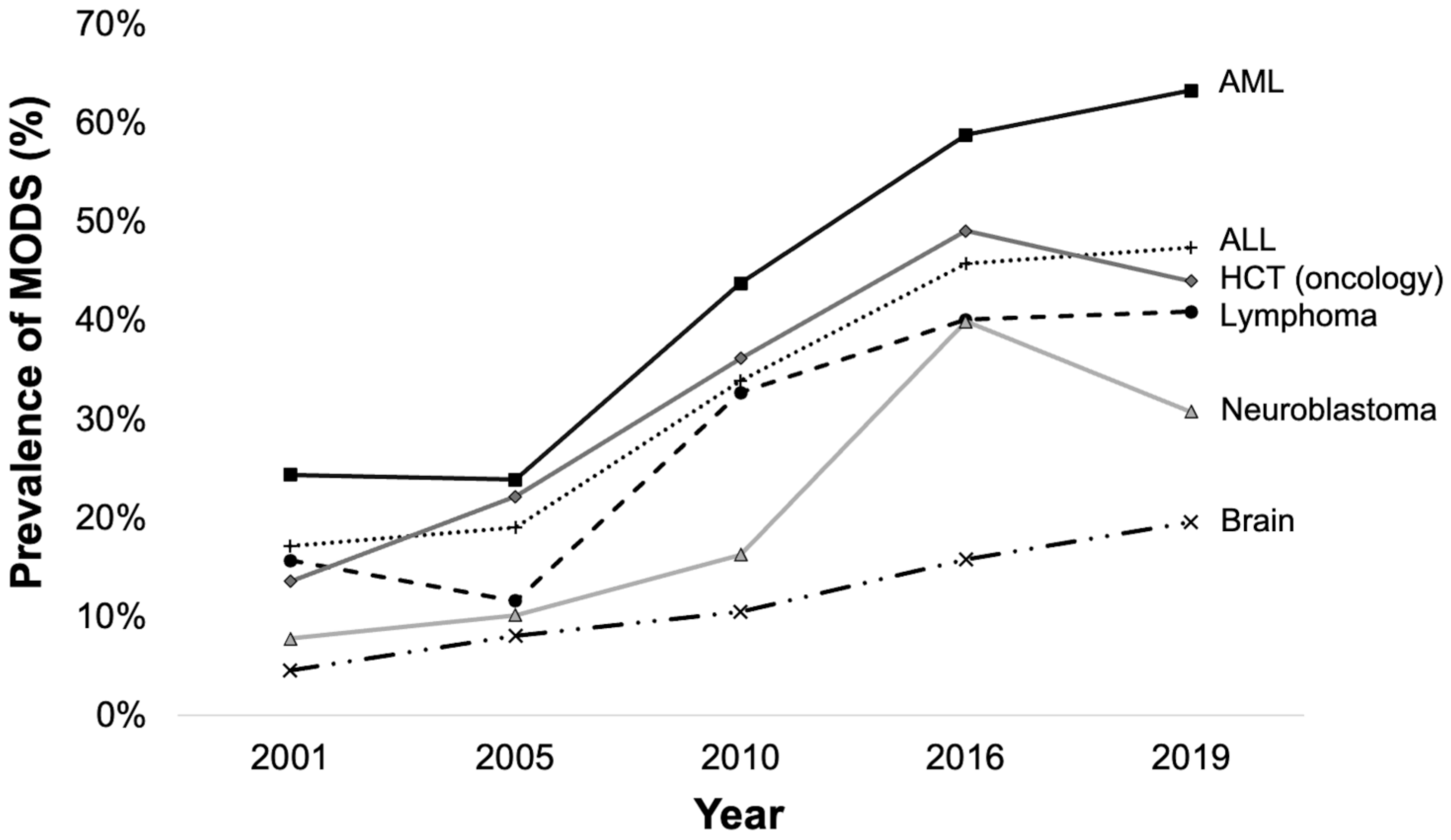

eFigure 1: prevalence of multiorgan dysfunction syndrome among intensive care unit pediatric patients by diagnosis.

ALL = acute lymphoblastic leukemia, AML = acute myeloid leukemia, HCT = hematopoietic stem cell transplant, MODS = Multiple Organ Dysfunction Syndrome.
